# An evaluation of distinct adeno-associated virus vector strategies for driving transgene expression in spinal inhibitory neurons of the rat

**DOI:** 10.3389/fnins.2025.1558581

**Published:** 2025-05-01

**Authors:** Laura Klinger, Anna S. M. Siegert, Raphael Holzinger, Lidia Trofimova, Sibel Ada, Ruth Drdla-Schutting

**Affiliations:** Division of Neurophysiology, Center for Brain Research, Medical University of Vienna, Vienna, Austria

**Keywords:** spinal cord, dorsal horn, inhibitory interneurons, nociception, adeno-associated virus vectors, intraparenchymal injection, rat

## Abstract

The spinal cord dorsal horn (DH) is essential for processing and transmitting nociceptive information. Its neuronal subpopulations exhibit significant heterogeneity in morphology and intrinsic properties, forming complex circuits that remain only partially understood. Under physiological and pathological conditions, inhibitory interneurons in the DH are of particular interest. These neurons modulate and refine pain-related signals entering the central nervous system. The ability to selectively target these inhibitory interneurons is key to investigating the underlying circuitry and mechanisms of pain processing, as well as to understand the specific role of inhibitory signaling within these processes. We employed a viral vector approach to deliver a fluorescent reporter protein specifically to inhibitory interneurons in the rat spinal cord. Using adeno-associated virus (AAV) vectors designed to express enhanced green fluorescent protein (EGFP) under the control of various promoters, we targeted distinct subtypes of spinal inhibitory interneurons. Through immunostaining, *in situ* hybridization, and confocal imaging, we evaluated the specificity and efficacy of these promoters. Our findings revealed that the promoter/vector combinations used did not achieve the desired specificity for targeting distinct interneuron populations in the DH. Despite these limitations, this work provides valuable insights into the potential and challenges of designing AAV-based approaches for selective neuronal targeting. These results emphasize the need for further refinement of promoter designs to achieve precise and reliable expression in specific spinal interneuron subtypes. Addressing these challenges will be crucial for advancing our understanding of spinal nociceptive circuits and developing targeted therapeutic approaches for pain syndromes.

## 1 Introduction

Acute pain is a vital physiological response that protects organisms from tissue damage. However, when pain persists beyond the initial cause, it can become pathological, significantly burdening patients. The mechanisms behind pathological pain are complex and often involve changes in pain processing circuits (Bravo et al., 2012; McWilliams et al., 2003). The spinal cord is the primary gateway for nociceptive signals, and within the dorsal horn (DH), inhibitory interneurons – comprising about 25–40% of all neurons in this region (Polgár et al., 2003; Todd and Sullivan, 1990) - play a crucial role in regulating pain signals before they are transmitted to the brain. Disruption of this inhibition can impair pain suppression, leading to various forms of pathological pain (Basbaum et al., 2009).

Electrophysiological measurements in acute tissue slices or *in vivo*, combined with advanced techniques like optogenetics, chemogenetics, and imaging, provide powerful tools for studying excitatory and inhibitory neuron interactions in nociception within the spinal cord. However, targeting inhibitory interneurons using conventional pharmacological methods is challenging due to their high heterogeneity (Stachowski and Dougherty, 2021). To overcome this, transgenic mice have been developed to express fluorescent reporter proteins in specific inhibitory neurons [e.g., (Bolneo et al., 2022; Lanuza et al., 2004; Madisen et al., 2010)]. Despite these advances, rats remain valuable in neuroscience and pain research due to their genetic similarity to humans, larger size, and the greater comparability of their nervous systems (Ellenbroek and Youn, 2016; Szpirer, 2020). This allows for more detailed investigation of specific neuronal pathways and cell types involved in nociception.

Although transgenic rat models have been developed over the past decade, their availability remains limited and they are not commonly used. Thus, alternative techniques like viral labeling to identify specific neuronal populations in rat studies are necessary (Qiu et al., 2022). Viral vectors, particularly adeno-associated virus (AAV) vectors, are powerful tools for gene transfer in preclinical research (Haggerty et al., 2019). AAVs are small, single-stranded DNA viruses with low pathogenicity that can infect various tissues and provide long-term gene expression. These vectors enable efficient analysis of neuronal circuits and manipulation of neurons *in situ* and *in vivo*. Specific AAV serotypes, such as AAV2 and AAV9, as well as pseudotyped variants, are particularly effective for gene delivery to neurons (Burger et al., 2004; Dayton et al., 2012; Howard et al., 2008; Jackson et al., 2015).

Glycine transporter 2 (GlyT2), Glutamate-Decarboxylase 67 (GAD67), and Paired box protein Pax-2 (Pax2), are commonly used markers of spinal inhibitory interneurons involved in nociceptive processing (Larsson, 2017; Mackie et al., 2003; Spike et al., 1997). Although the coding sequences of genes encoding these proteins are well-documented, identifying promoters that drive cell-type-specific expression, which is crucial for targeting specific cell types, remains challenging. The limited packaging capacity of AAV vectors (Ghosh et al., 2020) further complicates the inclusion of larger promoter sequences, particularly for genes that require long or complex regulatory regions to achieve precise expression. As a result, truncated or minimal promoter versions are often employed to fit within the vector’s capacity, which can compromise cell-type specificity and transgene expression strength, potentially leading to off-target expression or reduced activity (Taschenberger et al., 2017).

Here, we used AAV vectors with GlyT2, GAD67, and Pax2 promoters, which were either custom-designed or previously utilized in AAV vectors (Liu et al., 2013; Zhang et al., 2024), to drive enhanced green fluorescent protein (EGFP) expression in glycinergic, GABAergic, and broadly inhibitory interneurons within the rat spinal cord DH. However, none of the constructs tested achieved satisfactory specificity for transgene expression. This highlights the need for further optimization of promoters to enhance their utility in AAV vector-based approaches for investigating the role of spinal interneurons in nociceptive processing through electrophysiological, optogenetic, chemogenetic, and imaging experiments - both in acute tissue slices and *in vivo* - without reliance on time-intensive and costly transgenic models.

While our findings underscore the limitations of the current constructs, we believe this study provides a valuable contribution by identifying critical challenges in promoter design and laying the groundwork for the development of more precise targeting strategies in future research.

## 2 Methods

### 2.1 Animals and ethics declaration

Experiments were carried out using male Sprague–Dawley rats, at an experimental age of 21 to 24 days, which were bred in-house at the Medical University of Vienna, or purchased from Janvier Laboratories, Saint Berthevin Cedex, France. The rats were housed under standardized conditions including a 12-h light/dark cycle and free access to food and water. All procedures were performed according to the ARRIVE guidelines and in accordance with the European Communities Council directives (86/609/ EEC) and were approved by the Austrian Federal Ministry of Education, Science, and Research (BMBWF).

### 2.2 Spinal injection

Rats were anesthetized with isoflurane (5 vol% in O_2_ for induction, 3.5 vol% for maintenance) and ventilated via a face mask. Respiratory rate was maintained at 80–90 bpm, and temperature was controlled at 35–37°C using a rectal probe. After the loss of the tail pinch reflex, an incision was made, and a hemilaminectomy exposed the L4/L5 lumbar spinal cord. The spinal column was secured in a clamp mounted on a custom frame. Injections were performed as described elsewhere (Teuchmann et al., 2022). Briefly, a borosilicate glass pipette (30 μm tip) was used. The tip of the pipette was placed onto the spinal cord and positioned approximately 100 μm laterally to the central vein. The pipette was then inserted about 150 μm into the spinal cord from the tissue surface. 500 nL of either *AAV-2/9-{hSLC6A5_2kb + 5’UTRdelATG}-EGFP:WPRE* (1.54 × 10^12^ GC/mL), *AAV-2/9-{hSLC6A5_3kb + 5’UTRdelATG}-EGFP* (1.73 × 10^12^ GC/mL), *AAV-2/9-{hPAX2_1317bp}-EGFP:WPRE* (2.47 × 10^12^ GC/mL), obtained from VectorBuilder Inc. (Vector ID VB221213-1645dyw, VB221213-1650dya and VB221129-1069ytv, respectively) and *AAV-2/9-hGAD67-chI-EGFP-SV40p(A)* (4.1 × 10^12^ GC/mL) (Liu et al., 2013; Zhang et al., 2024), obtained from the Viral Vector Facility of the Neuroscience Center Zurich, were injected via a motorized microinjection pump with an injection speed of 50 nL·min^−1^. A second injection was made 1–2 mm away along the cranio-caudal axis. After injection, the rats were released from the clamp, anesthesia reduced to 2%, the incision sutured, and antibiotic ointment applied. Post-anesthesia, rats were housed singly and monitored until fully recovered. Carprofen (4 mg·kg^−1^ s.c.) was administered for analgesia. The next day, rats were reunited with their littermates in cages of 2–3.

### 2.3 Histological analysis

Animals were euthanized 15 to 21 days post the injection of viral vectors and transcardially perfused using 1% heparinized ice-cold saline solution, followed by 4% paraformaldehyde (PFA, 8.4 pH). Spinal cords were removed and post fixed overnight in PFA at 4°C after which they were cryoprotected in 20 and 30% sucrose in 0.1 M phosphate buffered saline (1x PBS) for 24 h, respectively, before being placed in an optimal cutting temperature compound (Sakura Finetek, Japan), flash-frozen in isopentane at −80°C and stored at the same temperature until further processing. Transversal slices (40 μm thick) were sliced with a cryostat and stored in well plates containing 0.05% Na-azide (Sigma Aldrich, S-2002) in 1x PBS at 4°C. For fluorescence *in situ* hybridization (FISH), the slices were thaw-mounted onto pre-cooled object slides and stored at −80°C.

Free-floating indirect immunohistochemical (IHC) stainings were performed on the 40 μm tissue slices. These were first washed in 1x PBS + 0.1% Triton X-100 (Merck, 3 × 10 min) and then incubated for 60 min in blocking solution: 1x PBS + 0.1% Triton X-100 and 4% normal goat serum (NGS, Cell Signaling Technology). All washing and incubation steps were carried out on a shaker (70 rmp) at room temperature. Primary antibodies (Pax2, Abcam ab150391, rabbit monoclonal, 1:150 dilution; NeuN, Millipore MAB377, mouse monoclonal, 1:600 dilution; calcitonin gene-related peptide (CGRP), Sigma C9487, mouse monoclonal, 1:1000 dilution; isolectin B4 (IB4), Vector B1205, biotin-conjugated, 1:200 dilution; protein kinase C gamma (PKCγp), Santa Cruz A0704, rabbit polyclonal, 1:500 dilution) were applied and incubated overnight. The slices were then washed in 1x PBS + 0.1% Triton X-100 (3 × 10 min) after which the secondary antibodies (Alexa Fluor 546, Thermo Fisher A11010, goat anti-rabbit, 1:1000 dilution; Alexa Fluor 647, Invitrogen A21235, goat anti-mouse, 1:1000 dilution; aminomethylcoumarin acetate (AMCA), Jackson 715–155-150, donkey anti-mouse, 1:500 dilution; Cy3, Jackson 016–160-084, streptavidin, 1:200 dilution; Cy5, Jackson 711–175-152, donkey anti-rabbit, 1:200 dilution) were added in blocking solution and incubated, guarded from light, for 2 h. Tissue slices were washed in 1x PBS (3 × 10 min), incubated in 1x PBS + 0.2% 40,6-diamidino-2-phenylindole (DAPI, Abcam, ab228549), guarded from light, for 60 min. Again slices were washed with 1x PBS (2 × 5 min) and mounted on glass slides (Superfrost plus, Thermo Fisher Scientific) and embedded with Fluoromount-G mounting medium (Thermo Fisher Scientific). Glass slides were stored at 4°C. 1 μm interval Z-stack images were obtained using an inverted confocal microscope (Zeiss LSM 780) to produce high resolution 3D images using the ZEN Software (ZEN 2.3 SP1). The hybridization chain reaction (HCR) kit v3.0 (Molecular Instruments) was used for RNA-FISH and performed according to the manufacturer’s instructions. Specifically, RNA probes targeted against GlyT2 (Slc6a5) and VGlut2 (Slc17a6) and corresponding HCR amplifiers conjugated to Alexa Fluor 546 and Alexa Fluor 647 were used. In the cases of Pax2 IHC combined with GlyT2-FISH, the FISH protocol was carried out first, followed by the IHC staining for Pax2 (Mussetto et al., 2023).

### 2.4 Data evaluation and statistics

The regions of interest (ROIs) included laminae I/II (LI/II), lamina III (LIII), and laminae IV/V (LIV/V). LI/II boundaries were defined using CGRP, PKCγ, and IB4, with CGRP marking the dorsal border and PKCγ and IB4 delineating the inner and outer layers of lamina II. Laminae III and IV/V were defined using anatomical landmarks. Within these ROIs, cells expressing markers such as NeuN or Pax2, as well as cells expressing EGFP, were automatically detected in their respective channels using the “Spot Detection” algorithm in IMARIS (version 9.6.0), with 2–3 slices analysed per animal. Co-labelled cells were identified using the “Shortest Distance to Spots” function with a predefined threshold of less than 6 μm. Both detected cells and algorithm-identified co-localizations were then manually inspected to ensure accuracy. Furthermore, DAPI staining - routinely performed in every section - was used to verify that each detected signal corresponded to a single cell rather than two overlapping cells being misinterpreted as one. The mean number of marker-positive cells, co-expression percentage, transduction efficacy, and viral spread were calculated using Excel. Data are expressed as mean ± standard error of the mean (SEM) and analyzed with GraphPad Prism 9.

## 3 Results

### 3.1 AAV-GlyT2 variants tested in the present study were not specific for glycinergic neurons in the spinal cord DH

**Figure 1:**
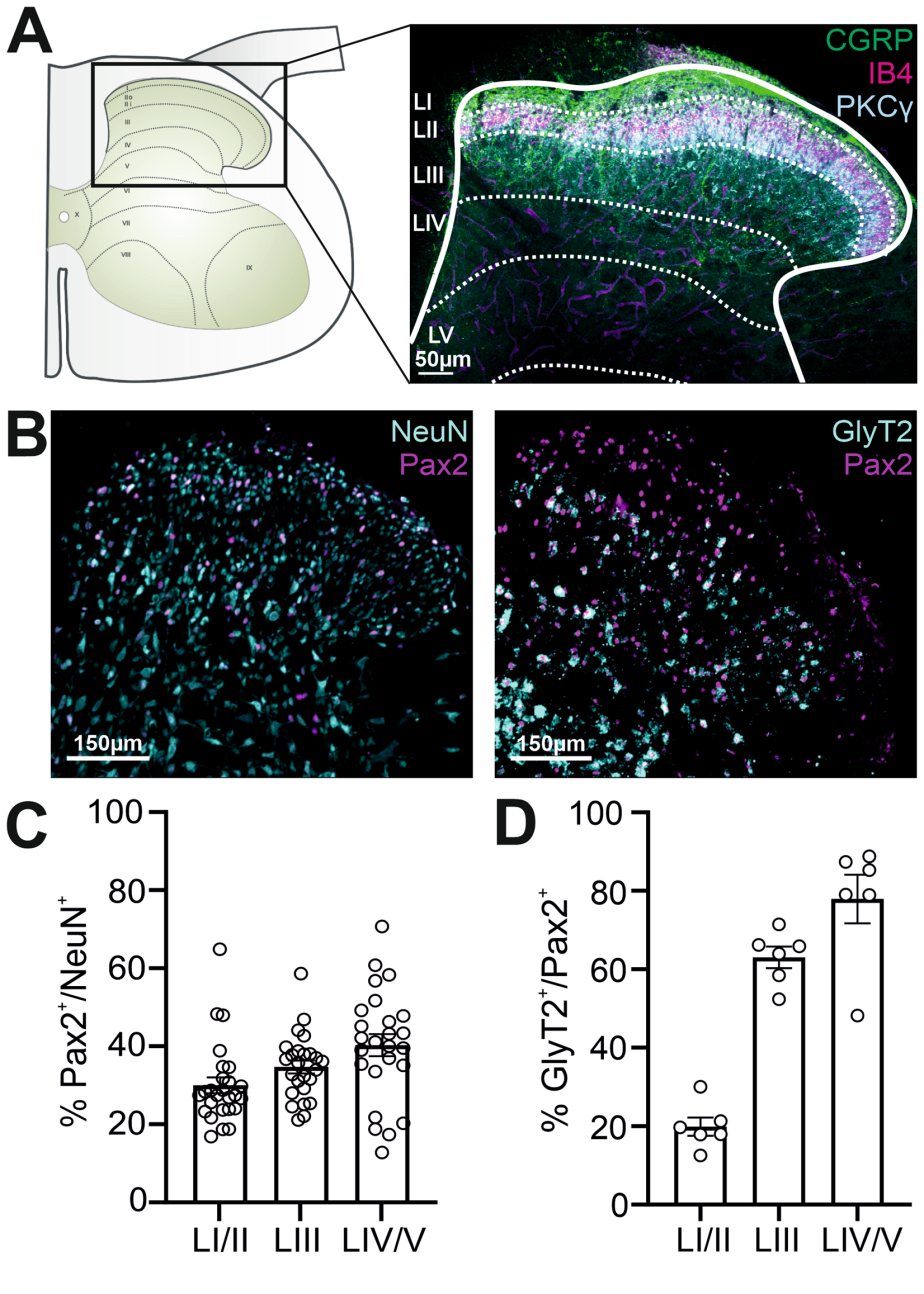
Laminar structure and distribution of inhibitory neurons identified by specific markers in the rat spinal cord DH. **(A)** A schematic (left) and confocal image (right) illustrate the laminar organization of the DH. Laminae I and II (LI/II) are identified by CGRP^+^ fibers (green), IB4^+^ fibers (magenta), and PKCγ^+^ interneurons (cyan). Lamina III (LIII) is defined by specific anatomical landmarks, while Laminae IV and V (LIV/V) are located caudal to LIII and delimited by anatomical landmarks. The dotted line delineates the boundaries of the spinal DH laminae. **(B)** Left: Slices were stained with antibodies against NeuN (cyan) and Pax2 (magenta); right: FISH was performed with probes for GlyT2 (cyan) and combined with immunostaining for Pax2 (magenta). **(C)** Percentage of Pax2^+^ cells among all NeuN^+^ cells across distinct laminae; n = 26 slices from a total of 9 animals. **(D)** Percentage of GlyT2^+^ cells among all Pax2-expressing cells across distinct laminae; n = 6 slices from 3 animals. All data are shown as mean ± SEM.

Proper nociceptive processing in the DH depends on a balance of excitatory and inhibitory activity. To quantify inhibitory neurons in specific DH laminae (Figure 1A), we stained rat tissue sections with NeuN (neuronal marker) and Pax2, which labels both glycinergic and GABAergic neurons (Miranda et al., 2023) (Figure 1B). We found inhibitory neurons constituted 30 ± 2% in LI/II, 35 ± 2% in LIII, and 40 ± 3% in LIV/V, consistent with reported values (Todd and Sullivan, 1990) (Figure 1C). Although both GABAergic and glycinergic neurons play pivotal roles in regulating nociceptive processing, the glycinergic component predominates under physiological conditions (Foster et al., 2015). Here, glycinergic neurons were identified by GlyT2 (Slc6a5) expression (Spike et al., 1997). Using FISH for GlyT2 mRNA combined with immunostainings, we found 20 ± 2% of Pax2^+^ cells in LI/II, 63 ± 3% in LIII, and 78 ± 6% in LIV/V were glycinergic (Figure 1D).

**Figure 2:**
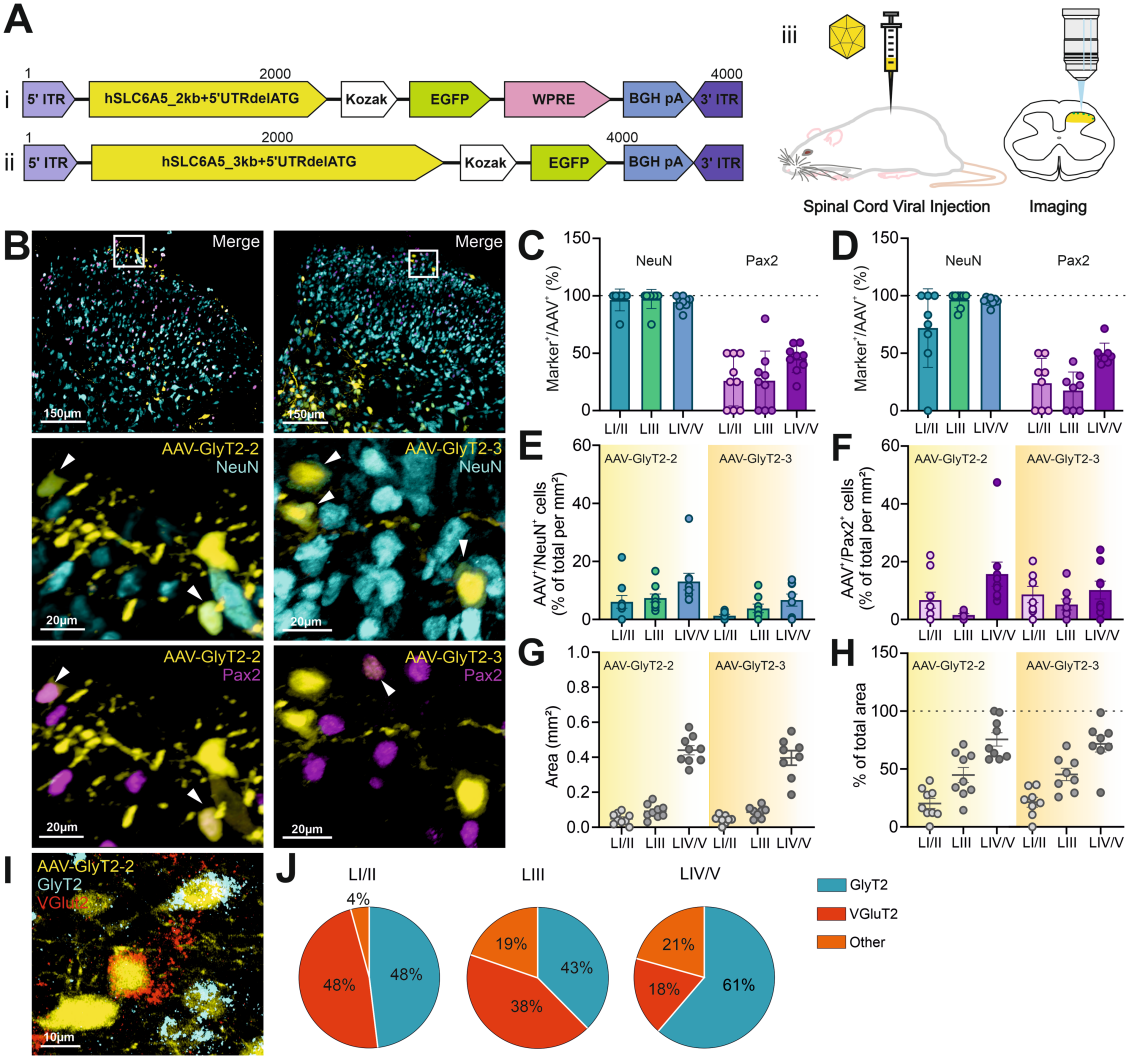
AAV-GlyT2-mediated transgene expression was not restricted to inhibitory neurons. **(A)** Schematic of the AAV constructs showing the insertion of hSLC6A5_2kb **(i)**, or hSLC6A5_3kb **(ii)**, downstream of the AAV 5′ inverted terminal repeat (ITR). Vectors were injected into the spinal cord DH **(iii)**, and viral expression was assessed 15–21 days post-injection. **(B)** Representative confocal images displaying an overview of AAV-GlyT2-2 (right) and AAV-GlyT2-3 (left), with magnified views of an area in LI/II marked by the white box; EGFP is shown in yellow, NeuN in cyan, and Pax2 in magenta. Arrows indicate co-localized cells. **(C, D)** Proportion of AAV^+^ cells in each lamina co-expressing either NeuN or Pax2 for AAV-GlyT2-2 (C) and AAV-GlyT2-3 **(D)**. **(E,F)** Viral transduction efficiency, shown as the percentage of EGFP-expressing cells per mm^2^ within NeuN^+^ **(E)** or Pax2^+^ **(F)** cell populations for each AAV-vector. **(G)** Viral spread across specific laminae, quantified in mm^2^ for AAV-GlyT2-2 and AAV-GlyT2-3. **(H)** Viral spread expressed as a percentage of the total area within each respective region of interest. Data show mean ± SEM. AAV-GlyT2-2: n = 9 slices from 3 animals; AAVGlyT2-3: n = 8 slices from 3 animals; data were compared using unpaired t-Tests. No significant differences were detected between both AAV-vectors. **(I)** GlyT2 (cyan) and VGlut2 (red) were detected by FISH. **(J)** Pie charts represent the percentage of AAV^+^ cells co-localized with either GlyT2 or VGlut2: n = 8 slices from 3 animals.

To study these neurons in the rat spinal cord without relying on genetic manipulations, we aimed to target them using a fragmented GlyT2 promoter to drive the expression of EGFP, which was packaged into an AAV2/9 and injected into the DH of the spinal cord of rats (Figure 2A). Although the sequence of the GlyT2 gene is known, the limited packaging capacity of AAVs prevents the use of the full-length promoter. In this study, we chose to start by using a 2000 bp sequence upstream of the transcription start site (TSS), packed in a viral vector, and tested its efficacy in driving EGFP expression in glycinergic cells (Figures 2A,i).

We observed robust expression of the EGFP reporter protein following the injection of AAV-2/9-{hSLC6A5_2kb + 5’UTRdelATG}-EGFP (referred to as AAV-GlyT2-2) into the spinal cord DH, with a distribution pattern favoring deeper laminae (Figure 2B). NeuN immunostaining confirmed that most EGFP-positive cells were neurons, with co-labeling rates of 96 ± 4% in LI/II, 97 ± 3% in LIII, and 95 ± 2% in LIV/V (Figure 2C). Co-staining with Pax2 indicated that 26–45% of these neurons were inhibitory (LI/II: 26 ± 7%; LIII: 26 ± 9%; LIV/V: 45 ± 4%; Figure 2C).

To enhance specificity for glycinergic neurons, we tested a modified viral construct with a 3000 bp sequence upstream of the TSS (AAV-2/9-{hSLC6A5_3kb + 5’UTRdelATG}-EGFP; AAV-GlyT2-3; Figures 2A,ii). Immunostainings revealed that 72 ± 12% of transduced cells in LI/II, 97 ± 2% in LIII, and 96 ± 1% in LIV/V were co-labeled with NeuN (Figure 2D). However, Pax2 co-labeling again indicated low proportions of inhibitory neurons (LI/II: 24 ± 8%; LIII: 18 ± 6%; LIV/V: 49 ± 3%; Figure 2D).

To evaluate viral transduction efficiency, we calculated the percentage of neurons transduced per unit area (mm^2^). For AAV-GlyT2-2, the transduction efficiency was 6 ± 2% in LI/II, 7 ± 1% in LIII, and 13 ± 3% in LIV/V. Comparable results were obtained with AAV-GlyT2-3, with transduction efficiencies of 1 ± 0.5% in LI/II, 4 ± 2% in LIII, and 7 ± 2% in LIV/V. Unpaired t-tests comparing AAV-GlyT2-2 and AAV-GlyT2-3 showed no significant differences in this parameter (LI/II: *p* = 0.081; LIII: *p* = 0.1; LIV/V: *p* = 0.1; Figure 2E). The transduction efficiencies of Pax2-positive cells (percentage of Pax2-positive neurons per mm^2^) for AAV-GlyT2-2 were as follows: 7 ± 3% in LI/II, 2 ± 0.4% in LIII, and 16 ± 4% in LIV/V. For AAV-GlyT2-3, the transduction efficiencies were 9 ± 3% in LI/II, 5 ± 2% in LIII, and 10 ± 3% in LIV/V. There were no statistically significant differences between AAV-GlyT2-2 and AAV-GlyT2-3 (unpaired t-tests; LI/II: *p* = 0.65; LIII: *p* = 0.07; LIV/V: *p* = 0.3; Figure 2F).

Due to space constraints, the WPRE [Woodchuck Hepatitis Virus Posttranscriptional Regulatory Element, commonly used to enhance viral vector expression (Lee et al., 2005)] was omitted in the AAV-GlyT2-3 construct. Despite this, viral spread was comparable to that achieved with the previous construct. For AAV-GlyT2-3, the transduced area was LI/II: 0.04 ± 0.01 mm^2^, LIII: 0.09 ± 0.01 mm^2^, LIV/V: 0.4 ± 0.04 mm^2^, with the percentage of total area being LI/II: 20 ± 4%, LIII: 45 ± 7%, LIV/V: 73 ± 6%. In comparison, AAV-GlyT2-2 had an area of LI/II: 0.05 ± 0.01 mm^2^, LIII: 0.09 ± 0.01 mm^2^, LIV/V: 0.44 ± 0.03 mm^2^, with percentages of total area being LI/II: 21 ± 4%, LIII: 45 ± 5%, LIV/V: 70 ± 7%. Unpaired *t*-tests between AAV-GlyT2-2 and AAV-GlyT2-3 showed no significant differences for area (LI/II: *p* = 0.92; LIII: *p* = 0.97; LIV/V: *p* = 0.8) or percentage of total area (LI/II: *p* = 0.74; LIII: *p* = 0.8; LIV/V: *p* = 0.37) (Figures 2G,H).

To further refine our analysis, we performed FISH to assess mRNA expression in EGFP-expressing cells transduced by AAV-GlyT2-2. A subset of these cells expressed GlyT2 mRNA, identifying them as glycinergic (LI/II: 48 ± 16%; LIII: 38 ± 12%; LIV/V: 61 ± 6%). Other EGFP-positive cells expressed vesicular glutamate transporter (VGlut2) mRNA, marking them as excitatory (Llewellyn-Smith et al., 2007; Zhou et al., 2007) (LI/II: 48 ± 16%; LIII: 43 ± 11%; LIV/V: 18 ± 4%). Another population of EGFP-positive cells lacked both GlyT2 and VGlut2 expression (LI/II: 4 ± 3%; LIII: 19 ± 6%; LIV/V: 21 ± 5%; Figures 2I,J).

### 3.2 Targeting GABAergic neurons using an AAV-GAD67 construct

**Figure 3:**
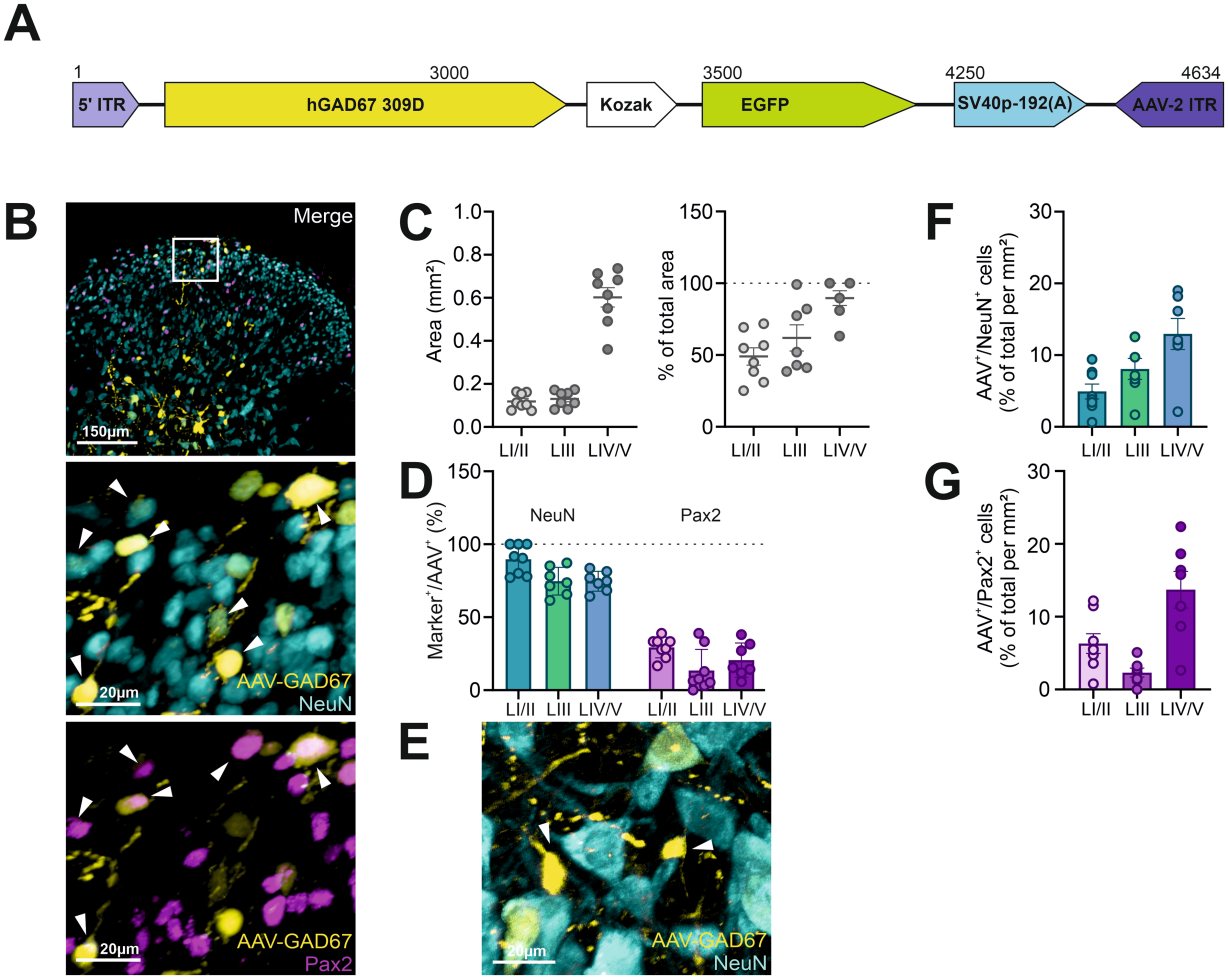
The AAV-GAD67 construct used in this study transfected neuronal and non-neuronal cells. **(A)** Diagram of the AAV construct used. **(B)** Representative confocal images illustrating AAV-GAD67 expression (EGFP, yellow) in combination with NeuN (cyan) and Pax2 (magenta). Enlarged views of the region marked by the white box in the main image are shown in the middle and lower panel, with arrows highlighting co-localized cells. **(C)** Distribution of viral expression across individual laminae, represented in mm^2^ (left) and as a percentage of the total area of each defined region (right). **(D)** Fraction of AAV^+^ cells within each lamina co-expressing NeuN or Pax2. **(E)** Representative image of AAV-GAD67 transfected cells (yellow) without NeuN (cyan) co-labeling (arrow). **(F,G)** Transduction efficiency, presented as the percentage of EGFP^+^ cells per mm^2^ relative to the total NeuN^+^ **(F)** or Pax2^+^ **(G)** populations. *n* = 7 to 8 slices from 3 animals. Data show mean ± SEM.

We next tested an AAV that was designed to drive the expression of EGFP under a fragment of the human GAD67 promoter, which has been successfully used to target GABAergic cells in the cortex (Liu et al., 2013) and ventral tegmental area (Zhang et al., 2024). In the spinal cord, injection of AAV-2/9-hGAD67-chI-EGFP-SV40p(A) (referred to as AAV-GAD67; Figures 3A,B) resulted in a viral spread area of 0.2 ± 0.01 mm^2^ in LI/II, 0.13 ± 0.01 mm^2^ in LIII and 0.6 ± 0.05 mm^2^ in LIV/V, with percentage of total area being: LI/LII: 49 ± 6%, LIII: 62 ± 9%, LIV/V: 86 ± 6% (Figure 3C). This construct primarily drove EGFP expression in neurons, with 90 ± 4% in LI/II, 75 ± 4% in LIII, and 75 ± 3% in LIV/V co-expressing NeuN (Figure 3D).

However, only a relatively small percentage of EGFP-expressing cells were found to be also Pax2-positive (LI/II: 29 ± 3%; LIII: 13 ± 5%; LIV/V: 21 ± 5%; Figure 3D), and expression in NeuN-negative cells was frequently observed, particularly in the deeper laminae (Figure 3E). Transduction efficiency was relatively low for this AAV, with an average of 5 ± 1% of all NeuN-positive cells co-expressing EGFP in LI, 8 ± 1% in LIII, and 13 ± 2% in LIV/V per mm^2^ (Figure 3F). This makes this AAV quite ineffective. In contrast, 6 ± 1% of Pax2-positive cells co-expressed EGFP in LI/II, 2 ± 1% in LIII, and 14 ± 3% in LIV/V per mm^2^, which is similar to the results obtained with AAV-GlyT2-2 (Figure 3G).

### 3.3 Spinal inhibitory neurons were not effectively transduced by an AAV-Pax2 construct

**Figure 4:**
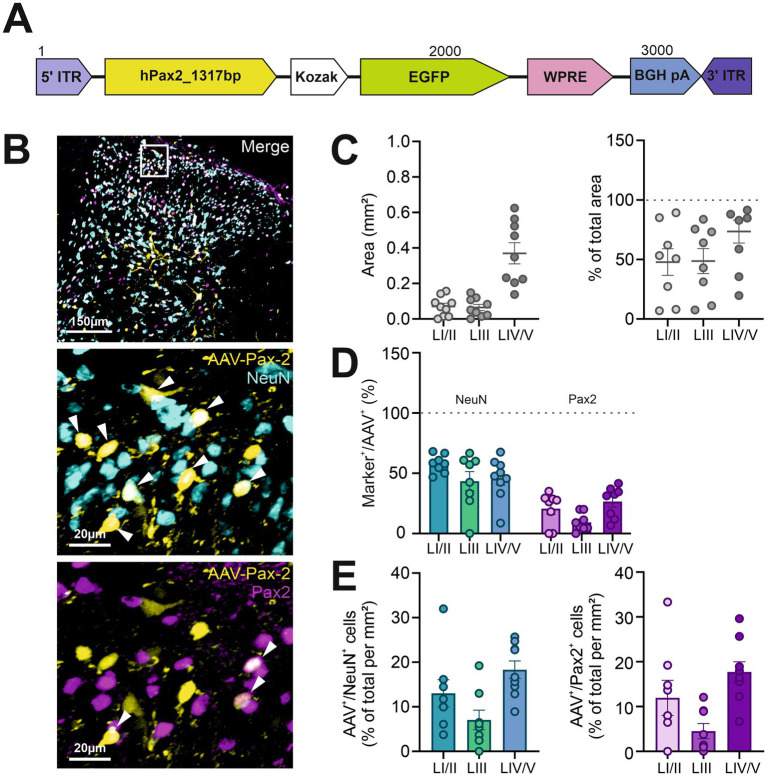
The AAV-Pax2 vector used in the present study did not specifically transduce spinal inhibitory neurons. **(A)** Scheme of the AAV construct used. **(B)** Representative confocal images showing AAV-Pax2 expression (EGFP, yellow) alongside NeuN (cyan) and Pax2 (magenta). Enlarged views of the area outlined by the white box in the first image are presented in the middle and lower panels, with arrows indicating co-localized cells. **(C)** Distribution of viral expression across individual laminae, displayed in mm^2^ (left) and as a percentage of the total area in each specified region (right). **(D)** Proportion of AAV + cells within each lamina co-expressing NeuN or Pax2. **(E)** Transduction efficiency, shown as the percentage of EGFP^+^ cells per mm^2^ relative to the total NeuN^+^ (left) or Pax2^+^ (right) populations. *n* = 8 to 9 slices from 3 animals. Data show mean ± SEM.

Finally, we aimed to target EGFP expression to inhibitory neurons by using a Pax2 promoter fragment (AAV-2/9-{hPAX2_1317bp}-EGFP:WPRE; AAV-Pax2) (Figure 4A). We injected this vector into the DH to evaluate this 1317 bp promoter, previously shown to have strong transcriptional activity (Stayner et al., 1998), for its ability to drive EGFP expression in spinal inhibitory neurons (Figure 4B). Again, the viral spread showed a distinct distribution between the different laminae, with a higher coverage in deeper laminae (area: LI/II: 0.1 ± 0.02 mm^2^; LIII: 0.1 ± 0.02 mm^2^; LIV/V: 0.4 ± 0.1 mm^2^, with percentage of total area LI/II: 48 ± 11%; LIII: 49 ± 11%; LIV/V: 66 ± 11%) (Figure 4C).

We found that, on average, between 46 and 58% of EGFP-positive cells were co-labelled with NeuN (LI/II: 58 ± 3%; LIII: 44 ± 8%; LIV/V: 46 ± 6%), while the remaining cells were NeuN-negative, suggesting they are likely non-neuronal. Immunostainings further revealed that 21 ± 5% in LI/II, 9 ± 3% in LIII, and 27 ± 5% in LIV/V of cells expressing EGFP were co-labelled with Pax2 antibodies (Figure 4D). Although AAV-Pax2 displayed low tropism for neurons, its transduction efficiency was generally comparable to or slightly better than that of the other AAV vectors tested, with 13 ± 3% of neurons per mm^2^ in LI/II, 7 ± 2% in LIII, and 18 ± 2% in LIV/V expressing EGFP, and 12 ± 4% of all Pax2-positive cells per mm^2^ in LI/II, 5 ± 2% in LIII and 18 ± 2% in LIV/V expressing the fluorescent reporter protein (Figure 4E).

## 4 Discussion

Studying nociceptive processing in the human spinal cord is constrained by ethical and practical limitations, leading to reliance on surrogate models. While transgenic models have driven significant discoveries, their use is costly, labor-intensive, and currently still limited in rats. Consequently, viral strategies, particularly AAV vectors, are increasingly employed to target specific neuronal populations. AAVs enable precise control of gene expression in neurons through tailored serotypes and promoters, making them invaluable for investigating distinct cell types (Flint et al., 2015; Lau and Suh, 2017; Wang et al., 2019).

Previous studies have used promoters like calcium-calmodulin-dependent protein kinase II *α* (CaMKIIα) and human synapsin (hSyn1) to target spinal cord neurons, with hSyn1 broadly targeting all neurons and CaMKIIα focusing on excitatory neurons, though its specificity is debated (Kaur and Berg, 2022; Larsson, 2018; Veres et al., 2023; Watakabe et al., 2015). Targeting inhibitory interneurons has largely relied on transgenic mice. Recently, an AAV2-mDlx enhancer successfully targeted GABAergic neurons in the rat spinal cord but was less effective in the superficial DH, crucial for nociceptive processing, and also induced expression in glutamatergic neurons (Kaur and Berg, 2022). These limitations make the approach unsuitable for many studies focused on nociceptive processing. Promoters for GlyT2, GAD67, and Pax2, key markers of spinal inhibitory interneurons, may offer a more precise approach for AAV-mediated transgene expression.

GlyT2 is a neuron-specific cell membrane transporter responsible for the uptake of glycine from the synaptic cleft (Spike et al., 1997). It is expressed in spinal glycinergic interneurons, and enhancing inhibitory neurotransmission through the inhibition of this transporter represents a promising therapeutic approach for treating neuropathic pain (Vandenberg et al., 2014). Glycinergic neurons have been successfully labeled in mice *in vivo* by expression of a green fluorescent protein under the control of the endogenous promoter derived from the GlyT2 gene SLC65A (Zeilhofer et al., 2005), and GLYT2-Cre transgenic mouse lines have been developed using bacterial artificial chromosomes, which contain more than 100 kb DNA sequence (Kakizaki et al., 2017; Rahman et al., 2015).

Since AAV vectors have a packaging capacity of less than 5 kb, promoters must be compact to accommodate relatively larger transgenes. A previous study identified a basal promoter within 304 bp upstream of the TSS (Borowsky and Hoffman, 1998), but its activity in nervous tissue has not been thoroughly explored. In this study, we tested a 2000 bp sequence upstream of the TSS as a promoter; however, this did not yield the expected expression of EGFP in glycinergic neurons. While most of the transduced cells were indeed neurons, only a small percentage expressed the inhibitory marker protein Pax2 or GlyT2 mRNA. In contrast, a large proportion of cells were VGlut2-positive, as revealed by FISH.

Similarly, using a longer sequence spanning 3000 bp upstream of the TSS did not improve specificity. It is possible that including longer sequences reduces specificity by introducing additional regulatory elements or transcription factor binding sites that alter promoter function (Tuğrul et al., 2015). Interestingly, omitting the WPRE - generally considered critical for strong transgene expression (Lee et al., 2005) - had no discernible effect under our experimental conditions.

GAD67 is one of two forms of glutamate decarboxylase, an enzyme required for GABA synthesis, and is abundantly expressed throughout the spinal gray matter, including the DH (Mackie et al., 2003). A 3100 bp fragment of the GAD67 (GAD1) promoter has been successfully used in AAVs to drive transgene expression in GABAergic neurons in the brain (Zhang et al., 2024). Using essentially the same construct as in the study by Zhang and colleagues, but with EGFP instead of mScarlet and the same promoter to drive EGFP expression, did not result in high specificity for inhibitory cells in the spinal cord. This demonstrates the significant variability that can occur between species and regions due to differences in transcription factor expression and regulatory networks.

In another attempt to target spinal inhibitory neurons, we tested the ability of a Pax2 promoter to drive transgene expression. Pax2 is a transcription factor persistently expressed into adulthood and is commonly used as a general marker of inhibitory neurons (Burrill et al., 1997; Larsson, 2017). The human Pax2 promoter was originally identified in fetal kidney and Wilm’s tumor (Stayner et al., 1998), where a 1317 bp sequence demonstrated high transcriptional activity in various cell lines. We used this sequence to drive EGFP expression in Pax2-expressing, i.e., inhibitory, spinal neurons. However, the expression observed was once again not specific, suggesting that further modifications may be necessary to achieve selective targeting in the spinal cord.

To improve the specificity of transgene expression, vectors with larger packaging capacities, such as “high-capacity” adenoviral vectors - which can accommodate up to 36 kb of genetic material (Alba et al., 2005) - or lentiviral vectors could be considered. However, these tools come with limitations: they require viral proteins to be supplied in trans by a helper virus (Alba et al., 2005), elicit stronger immune responses (Zaiss et al., 2002), and necessitate higher biosafety precautions, making them more difficult to use in a standard laboratory setting. In contrast, AAV vectors, while offering a smaller packaging capacity, are easier to handle and have a better safety profile.

The specificity of expression using AAVs depends not only on the promoter but also on the viral serotype. In this study, we employed pseudotyped AAV vectors by packaging AAV2-based genomes in AAV9 capsids. Although this approach has previously demonstrated high transduction efficiency in neurons and astrocytes in the spinal cord (Bucher et al., 2014; Dayton et al., 2012; Mussetto et al., 2023; Siu et al., 2017; Zhang et al., 2024), the transduction efficiency observed in the present study was unexpectedly low. All tested AAVs resulted in the transduction of only a small proportion of neurons and inhibitory cells within the regions of interest.

AAV-mediated gene delivery remains the gold standard for targeting specific cell types in the rat central nervous system, despite its limitations. In this study, we explored four different promoters to target inhibitory interneurons in the spinal cord DH of rats. However, none of the constructs tested achieved sufficient specificity and therefore cannot be used to selectively label these neurons. While promoters can be further optimized, and alternative serotype/promoter combinations might improve specificity and transduction efficiency, these refinements were beyond the scope of our study.

Our findings with constructs previously used successfully in other contexts (i.e., AAV-GAD67) also underscore the significant regional variability in promoter specificity within the central nervous system (CNS). This highlights the necessity of rigorous control experiments when using AAV tools - particularly when adapting them for use in different species or different CNS regions.

Although our approach did not achieve selective targeting of inhibitory interneurons in the spinal cord DH, our study provides valuable insights that may guide the development of more refined strategies to improve cell-type specificity. By addressing the challenges and variability associated with promoter-driven transgene expression, our findings contribute to the ongoing efforts to enhance the reliability and reproducibility of experimental approaches in neuroscience.

## Data Availability

The original contributions presented in the study are included in the article/supplementary material, further inquiries can be directed to the corresponding author.
